# A placebo-controlled study comparing the efficacy of intra-articular injections of hyaluronic acid and a novel hyaluronic acid-platelet-rich plasma conjugate in a canine model of osteoarthritis

**DOI:** 10.1186/s13018-019-1352-1

**Published:** 2019-09-18

**Authors:** Mun-Ik Lee, Jun-Hyung Kim, Ho-Hyun Kwak, Heung-Myong Woo, Jeong-Hee Han, Avner Yayon, Yun-Chan Jung, Jin-Man Cho, Byung-Jae Kang

**Affiliations:** 1 College of Veterinary Medicine and Institute of Veterinary Science, Kangwon National University, Chuncheon, 24341 Korea; 2 ProCore-biomed, Ltd., Weizmann Science Park, 76400 Ness Ziona, Israel; 3 Chaon, Seongnam, 13493 Korea; 4 Research Institute, Green Cross Veterinary Products Co., Ltd., Yongin, 17066 Korea; 5 Department of Veterinary Clinical Sciences, College of Veterinary Medicine and Research Institute for Veterinary Science, Seoul National University, 1Gwanak-ro, Seoul, Gwanak-gu 08826 Korea

**Keywords:** Dog, Hyaluronic acid, Hyaluronic acid conjugated with autologous fibrinogen from platelet-rich plasma, Osteoarthritis, Stifle joint

## Abstract

**Background:**

The objective of this study was to assess the efficacy of intra-articular injections of hyaluronic acid (HA) and a novel, on-site conjugate of HA with autologous fibrinogen in platelet-rich plasma (HA-PRP) in a canine model of osteoarthritis (OA)

**Methods:**

Twelve beagle dogs underwent a unilateral resection of the cranial cruciate ligament (CrCL) of the stifle joint. Clinical and radiographic signs of OA were confirmed in all dogs 8 weeks following CrCL resection and prior to treatment. The dogs were randomized into three groups: saline (*n* = 4), HA (*n* = 4), and HA-PRP (*n* = 4). Each dog received intra-articular injections of the respective substance into the affected joint at pre-determined time points. The dogs were assessed for adverse effects for 3 days after each injection and for lameness, pain, range of motion, kinetics, and radiographic OA severity prior to treatment and 3 months after injection. OA severity as determined by radiographic examination was not significantly different among the groups at any time point. The dogs were then humanely euthanatized and the stifle joint assessed by gross and histological examinations.

**Results:**

Dogs treated with four weekly injections of HA or two biweekly injections of HA-PRP were significantly (*p* < 0.05) better than dogs treated with four weekly injections of saline at 2-, 4-, and 12-week time points based on a comfortable range of motion (CROM) and clinical lameness score. Gait analysis measuring symmetry and weight distribution on pressure sensor walkway showed significantly (*p* < 0.05) improved limb function for dogs treated with HA and HA-PRP compared with dogs treated with saline yet with better clinical outcome for the HA-PRP-treated group at 12 and 20 weeks follow-up. Gross and histological analysis of synovium and articular cartilage demonstrated significant (*p* < 0.05) improvement by both treatments groups compared to controls. There was however significantly (*p* < 0.05) less damage to the cartilage in the HA-PRP group compared to the HA-treated group.

**Conclusions:**

These data suggest that while injection of HA and HA-PRP may be sufficient for short-term amelioration of the symptoms associated with OA, treatment with HA-PRP conjugates may be superior, providing significantly better long-term cartilage preservation.

## Background

Osteoarthritis (OA) is a debilitating joint disease, often secondary to structural abnormalities or ligament injury leading to articular instability and transformations of the normal cartilage matrix resulting in pain, stiffness of joints, and muscle atrophy [1, 2]. OA is the most common type of arthritis related to age and currently defined as an incurable disease. Moreover, the inflammatory process can affect the progression of the disease [3, 4].

The most important goal of treatments for osteoarthritis is to alleviate pain. Pain management allows the patient to regain strength and function and maintain the movability of the affected limb [5]. Other goals of treatment include the maintenance of joint flexibility, improvement of the patient’s quality of life, and potentially a delay in disease progression [6]. The most common methods for managing OA-related symptoms are the administration of non-steroidal anti-inflammatory drugs (NSAIDs), nutritional supplementation, physiotherapy, and weight management. The main adverse effects of NSAIDs are associated with the gastrointestinal tract, the kidney, and the impairment of platelet activity [7]. Alternatively, intra-articular injections of hyaluronic acid (HA) are gaining popularity in a number of countries as the first-line symptomatic treatment for diarthrodial osteoarthritis [8]. This treatment approach is based on the elastic properties of HA, increasing the lubricating properties similar to that of the synovial fluid in OA joints. Furthermore, HA can directly induce endogenous HA synthesis and has endogenous anti-inflammatory and antinociceptive properties [9, 10].

Platelet-rich plasma (PRP) is a concentrate of autologous platelets introduced recently also as an intra-articular agent to alleviate OA symptoms in animal and human medicine [11–15]. It is based on the intra-articular delivery of a large pool of growth factors and other bioactive proteins released from autologous platelet-rich preparations. Various growth factors (fibroblast growth factor [FGF], insulin-like growth factor [IGF], platelet-derived growth factor [PDGF], transforming growth factor-β [TGF-β], vascular endothelial growth factor [VEGF], etc.) released by the activated platelets serve critical roles in physiological processes such as wound healing and tissue regeneration. PRP contains not only platelets and growth factors but also fibrinogen, fibronectin, and other macromolecules that assist the healing process. Many of these growth factors and proteins have been found to take part also in the preservation and regeneration of articular cartilage, which given its avascular nature does not undergo spontaneous healing or regeneration. Last, platelets have also shown some analgesic and lubricating properties [16].

Although the interest in intra-articular viscosupplementation for the treatment of joint diseases is on the rise, it remains challenging mainly due to the rapid elimination of HA from the joint space through the synovial capillaries (for low-molecular HA) or the lymphatic vessels (for high-molecular HA) [17–19]. In addition, due to HA’s limited clinical efficacy, intra-articular drug delivery systems (DDS) are also gaining interest in joint regenerative medicine, with the objective of improving clinical and structural outcome [17, 19]. Hydrogels in particular have received significant attention as intra-articular drug delivery vehicle due to their biological and rheological properties, and high-molecular weight HA (HMW-HA) is a preferred biomaterial in the design of many of these hydrogels due to its physiological role in diarthrodial joint homeostasis and a key component of articular cartilage and synovial fluid [10, 20–23]. Another key component in our currently used hydrogel (Regenogel-OSP by Procore Ltd.) is fibrin, an autologous component and a product of fibrinogen derived from the patient’s own plasma. There have been previous reports demonstrating the affinity of HA to fibrin as well as their combination informing 3D scaffolds through covalent and ionic interactions [24–26] demonstrating improved mechanical properties and resistance against intra-articular deformation [27]. We hypothesized that autologous platelet-rich plasma conjugated with HMW-HA (HA-PRP) providing an authentic chemical conjugate made on site from the patient’s own fibrinogen and PRP and a pre-activated form of HMW-HA sharing enhanced stability, viscoelastic properties, and biocompatibility may result in a superior clinical and joint preservation outcome.

The objective this study was to compare the safety and efficacy of intra-articular injections of HA with that of HA-PRP, with saline as a control for symptomatic treatment of OA using the cranial cruciate ligament resection model in dogs. Functional methods of assessment included clinical lameness score (including the severity of pain), comfortable range of motion (CROM), and gait analysis. Radiographic, gross pathologic, and histologic assessments were also performed over the 20-week study period.

## Methods

Twelve adult beagle dog (2 years of age, body weight mean = 8.8 kg, range = 6.7–10 kg) purpose-bred research dogs were used. The dogs were permitted a 7-day acclimation period in the housing facilities prior to the initiation of the study.

### Preoperative assessments

Orthopedic examination by a trained veterinary orthopedic surgeon was performed on each dog before inclusion in the study (0 weeks). All limbs were evaluated to ensure that no pre-existing orthopedic disorders were evident. CROM was measured using a standard goniometer, as previously described [15, 28–30]. Clinical lameness scores were determined for each dog based on orthopedic examination by the same trained orthopedic clinician using modified criteria previously described [31] (Table 1). The total sum of the clinical lameness score was recorded for the statistical analysis.

**Table 1:** The criteria of clinical lameness score

Score criteria	
Score criteria	
[1] Normal stance	
[2] Slightly abnormal stance (partial weight-bearing of the limb, but the paw remains firmly in contact with floor)	
[3] Markedly abnormal stance (partial weight-bearing of the limb, with minimal contact between the paw and the floor)	
[4] Severely abnormal stance (no weight-bearing)	
Lameness at walk	
[1] No lameness; normal weight-bearing on all strides observed	
[2] Mild lameness with partial weight-bearing	
[3] Obvious lameness with partial weight-bearing	
[4] Marked lameness with no weight-bearing	
Lameness at trot	
[1] No lameness; normal weight-bearing on all strides observed	
[2] Mild lameness with partial weight-bearing	
[3] Obvious lameness with partial weight-bearing	
[4] Marked lameness with no weight-bearing	
Willingness to allow the clinician to lift the limb contralateral to the affected limb	
[1] Readily accepts contralateral limb elevation, bears full weight on the affected limb for more than 30 s	
[2] Offers mild resistance to contralateral limb elevation, bears full weight on the affected limb for more than 30 s	
[3] Offers moderate resistance to contralateral limb elevation and replaces it in less than 30 s	
[4] Offers strong resistance to elevation of contralateral limb and replaces it in less than 10 s	
[5] Refuses to raise contralateral limb	
Range of motion (ROM)	
[1] Full ROM	
[2] Mild decrease (10–20%), with no crepitus	
[3] Mild decrease (10–20%), with crepitus	
[4] Moderate decrease (20–50%)	
[5] Severe decrease (≥ 50%)	
Pain at palpation/mobilization	
[1] No pain elicited on palpation/mobilization of the affected joint	
[2] Mild pain elicited, e.g., turns the head in recognition	
[3] Moderate pain elicited, e.g., pulls the limb away	
[4] Severe pain elicited, e.g., vocalizes or becomes aggressive	
[5] Severe pain elicited, e.g., not allow examiner to palpate/mobilize the joint	
Evaluation of overall clinical condition	
[1] Good	
[2] Mildly poor	
[3] Moderately poor	
[4] Severely poor	
[5] Very severely poor	

### Cranial cruciate ligament (CrCL) resection

On the day of surgery, the dogs were pre-medicated, anesthetized, and prepared for aseptic surgical procedure of the right stifle using a hanging limb technique. After draping, each dog was positioned lateral recumbency, and a curved parapatellar skin incision was made in the right stifle joint. Another curved incision, similar to that in the skin, was preformed through the fascia lata along the cranial border of the biceps femoris. After separating the fascia from the joint capsule, a stab incision was made on the joint capsule and continued proximally and distally along with the fascia late incision line. The patella was retracted laterally, and the joint was opened. With the exposure of the joint, it was inspected that there was no evidence of pathological change in the intra-articular structures. With the opened joint fully flexed, the cranial cruciate ligament was identified and resected with the #11 blade. The joint capsule and fascia of the stifle joint were closed in one layer with 2-0 polydioxanone (PDS-II®, Johnson & Johnson International, USA). The subcutaneous tissue and skin were closed using 2-0 polydiaonanone (PDS-II®, Johnson & Johnson International, USA) and 2-0 polyamide (Nylon, AILEE, Korea).

Postoperative medications were injected with the tramadol (4 mg/kg, IV), meloxicam (0.2 mg/kg, IV), and the cefazolin (25 mg/kg, IV) for 72 h after surgery. All dogs were returned to their individual kennels and allowed unrestricted activity in the housing facility. In addition, all dogs were walked on a leash every day for 10–15 min at a pace to ensure use of all four limbs.

A physical examination was performed daily for the first 3 days after surgery and any observations recorded, including general condition, rectal temperature, appetite, and activity. The operated limb was observed daily for signs of swelling, erythema, heat, and dehiscence until suture removal (10 days after surgery). The dogs were managed in this way for 8 weeks prior to the treatment in order to establish chronic osteoarthritis in the CrCL-resected limb.

### Pre-treatment assessments

Eight weeks after CrCL resection (Pre), orthopedic examination to assess stifle CROM, knee pain, and clinical lameness score was performed on each dog. Kinetic assessment of the operated limb was performed using a pressure sensor walkway system (Tekscan, Inc., USA).

Craniocaudal and mediolateral radiographic views of the right stifle joint of each dog were obtained and scored by one trained veterinary clinician blinded to the treatment. The joints were examined for evidence of periarticular osteophyte formation and enthesophyte formation, subchondral bone sclerosis, articular margin irregularity, and subchondral bone cyst formation. Scores for the severity of osteoarthritic changes were assigned as follows: none (0), minimal (1), mild (2), moderate (3), or marked (4), as previously described for stifle joint [32]. The sum of radiographic OA severity score was recorded for statistical analysis.

### Preparation of injection materials

The following are the proposed procedures for preparing HA-PRP.

#### Preparation of PRP

Autologous PRP was prepared in each dog using double-spin method formerly described [33]. Thirty milliliters of fresh blood from each dog was collected in 10-mL tubes containing 3.2% sodium citrate to prevent coagulation. Before collecting the whole blood sample, 0.5 mL small aliquot of the whole blood was retained for initial complete cell count (CBC). Then, the blood sample was centrifuged at 1,000*g* for 5 min at room temperature. After the first centrifuge was completed, the upper plasma and platelet fraction (PRP1) were transferred into a sterile tube. The PRP1 fraction was centrifuged at 1,500*g* for 15 min at room temperature. Two thirds of the platelet-poor plasma (PPP) supernatant was drawn off, and the platelet-containing pellet PRP2 was resuspended in the remaining PPP. Through the above procedure, a total of 4 mL of PRP was obtained for each dog and used to make HA-PRP. Then, 0.5 mL aliquot of the obtained PRP was removed and compared to the initial CBC which should be at least four times the platelet counts of the initial CBC.

#### Preparation of HA-PRP

Inactivated powder of HA (Regenogel-OSP, Procore-biomed., Ltd., Israel) was used to make PRP-conjugated HA (HA-PRP). All procedures for the preparation of HA-PRP were followed by the manufacturer. A small volume (1.2 mL) of sterile water from the 10 mL water for injection ample was drawn and added into the inactivated powder of HA. The mixture of HA powder and sterile water was mixed using a shaker at 2.8*g* for 10 min at room temperature. Three milliliters of PRP obtained by the former procedure was mixed with the activated HA powder. Then, the HA-PRP solution was mixed at room temperature for 30 min using a shaker at about 2.8*g*. Then, all the HA-PRP mixtures were injected in each dog within 8 h.

### Intra-articular treatments

Under sedation and analgesia, the right stifle joint of each dog underwent aseptic arthrocentesis using a 23-gage needle and 3-mL syringe to remove the synovial fluid and ensure injections were intra-articular. The volume of the aspirated fluid was recorded. Each right stifle joint was then aseptically injected intra-articular space through the same needle used for arthrocentesis as follows:

- Control (saline) group: 2 mL of sterile 0.9% saline was injected weekly for a total of four injections beginning 8 weeks following CrCL resection.
- HA group: 2 mL of Hyaluronate® (Green Cross Veterinary Products Co., Ltd, Korea) was injected for a total of four injections beginning 8 weeks following CrCL resection.
- HA conjugated with PRP (HA-PRP) group: 2 mL of HA-PRP was injected once every 2 weeks (a total of two injections) beginning 8 weeks following CrCL resection.

All dogs recovered and returned to their individual kennels and allowed unrestricted activity in the housing facility. In addition, each dog was exercised on a leash every day for 10-15 min at pace to confirm use of all four limbs for the duration of the study.

### Post-treatment assessments

#### Evaluation of the adverse effects

All dogs underwent daily physical examination and palpation on the treated limb (evaluation of pain, heat, and swelling) during the intra-articular injection period. Blood works (CBC [Procyte DxTM, IDEXX Laboratories, USA], chemistry [Catalyst DxTM, IDEXX Laboratories, USA], and C-reactive protein [Catalyst DxTM, IDEXX Laboratories, USA]) for all dogs were performed at 3 days after the first intra-articular injection.

#### Kinetics assessment

At weeks 2, 4, and 12 after completion of the first treatment (10, 12, and 20 weeks after initiation of this study), kinetic assessment was recorded using a pressure sensor walkway (Tekscan, Inc., USA). All dogs were walked across the walkway system in one direction with the same examiner attempting to retain a consistent velocity on a loose leash. At least five acceptable passes (3–5 gait cycle), with video record, were acquired for each dog at each time point. Passes were included for analysis when the dogs walked at a regular pace with all four footprints recorded for at least five gait cycles. The Tekscan software (Tekscan, Inc., USA) was used to distinguish the paw print for each footfall, which was then identified manually as left front, left hind, right front, and right hind accordingly. In this way, at least five data points were collected for calculating symmetry index (SI) and weight distribution (WD) at each time point. The SI and WD were calculated using the following formulas: SI (%) = 100 − [(FI/Fc) × 100] (FI = parameter of the lame extremity and Fc = parameter of the of the contralateral extremity), WD (%) = the force-time integral (FTI, % BW × s) of the affected limb/sum of the FTI.

#### Orthopedic examination

Orthopedic examination to evaluate knee CROM, clinical lameness, and function assessments were performed on each dog at weeks 2, 4, and 12 after the completion of the first treatment. A single orthopedic clinician, blinded to the treatment, performed these analyses at all the time points.

#### Radiographic assessment

Under sedation with analgesia, radiographic assessments of the stifle joints were performed on each dog at weeks 12 after completion of the first treatment. A single trained clinician, blinded to treatment, analyzed all of the radiographic images based on the criteria previously described [32].

### Post-mortem assessments

At 20 weeks, after the completion of the first treatment, the dogs were humanely euthanatized. A full necropsy was performed immediately after euthanasia by three trained veterinary clinicians, who were blinded to the treatment group and clinical findings. The affected stifle joints from each dog were carefully dissected to evaluate gross pathology of the articular cartilage and synovium. Macroscopic alteration of the synovium (e.g., thickening [fibrosis], discoloration, and vascularity) and articular cartilage (e.g., cartilage structure, chondrocyte pathology, and proteoglycan staining) was scored using the scoring system set forth in the Osteoarthritis Research Society International (OARSI) histopathology initiative [34].

### Histologic assessments

After evaluating the macroscopic lesions of synovium and articular cartilage, portions of the synovial tissue were excised and fixed in formalin in the preparation for histologic processing.

The proximal part of the operated tibia and the distal part of the operated femur were excised and placed into 10% neutral buffered formalin. Bones were allowed to fix for 5 days and then placed in 10% EDTA. After decalcification was complete, the medial and lateral femoral condyles and medial and lateral tibial plateaus were each sliced into three sections approximately 2–4 mm thick for processing, embedding in paraffin, microtome sectioning (8 μm) and staining (Safranin O). Histologic scoring of the osteochondral tissues was performed by one veterinary clinician, blinded to the treatment, using the OARSI histologic scoring system for canine OA. Synovial tissue was routinely processed, sectioned (5 μm) and stained (hematoxylin and eosin [H&E]), and scored using the criteria proposed by the OARSI [34].

### Statistical analyses

The values were expressed as mean ± standard deviation (SD). Statistical analysis was performed using Graphpad Prism V 7.00 (Graphpad Software Inc., USA)

#### The CROM, clinical lameness score, and gait analysis

The repeated measures two-way analysis of variance (ANOVA) with post-test Tukey’s multiple comparison test was performed to assess the statistical significance for CROM, clinical lameness score, and gait analysis (SI, WD) within-group comparisons over time. Among the group, comparisons were done using two-way ANOVA with post-test Tukey’s multiple comparison test. Statistical significance was accepted for a value of *p* < 0.05.

#### Macroscopic and microscopic lesion assessments

The Kruskal-Wallis one-way ANOVA test was performed to assess the statistical significance of macroscopic and microscopic assessments. Furthermore, Dunn’s multiple comparison test was performed to evaluate the differences among the three groups. Statistical significance was accepted for a value of *p* < 0.05.

#### Radiographic assessment

The repeated measures two-way ANOVA with post-test Sidak’s multiple comparison test was performed to evaluate the statistical significance for intra-group OA score over time and inter-group OA score following the specific time points.

## Results

All 12 dogs successfully underwent CrCL resection, were assigned injection treatment, and survived for the intended period of the study.

### CrCL resection model for OA

No evidence for lameness or OA was present in any dog prior to CrCL resection. However, CrCL resection successfully induced clinical signs of lameness and OA by the time of intra-articular treatment (Table 2). Cranial draw motion and tibia compression test showed the ruptured cranial cruciate ligament and stifle joint instability. There were no significant differences among the groups with respect to the measures of kinetics, clinical lameness, CROM, and radiographic assessments at the time of treatment.

**Table 2:** Mean ± SD values for the outcome measures assessed before and after CrCL resection

	Lameness score	CROM (°)	SI (PVF, %)	SI (VI, %)	WD (PVF, %)	WD (VI, %)	X-ray OA
Pre-CrCL resection	1 ± 0	126 ± 4.3	4.5 ± 3.9	9.6 ± 6	19.9 ± 2	18.6 ± 2.3	0 ± 0
8 weeks after CrCL resection	17.75 ± 2.5	103 ± 4.9	81 ± 27.4	78.9 ± 37.9	10.9 ± 3.2	10.5 ± 4.1	7 ± 1.2

### Adverse events after intra-articular treatment

A dog (control [*n* = 1]) showed mild systemic inflammatory reaction based on the result of C-reactive protein. Two dogs (HA [*n* = 2]) showed non-weight-bearing lameness following intra-articular treatment. All the adverse events resolved within 3 days without the need for additional treatment.

### Clinical lameness score, CROM, and gait analysis

**Fig. 1:**
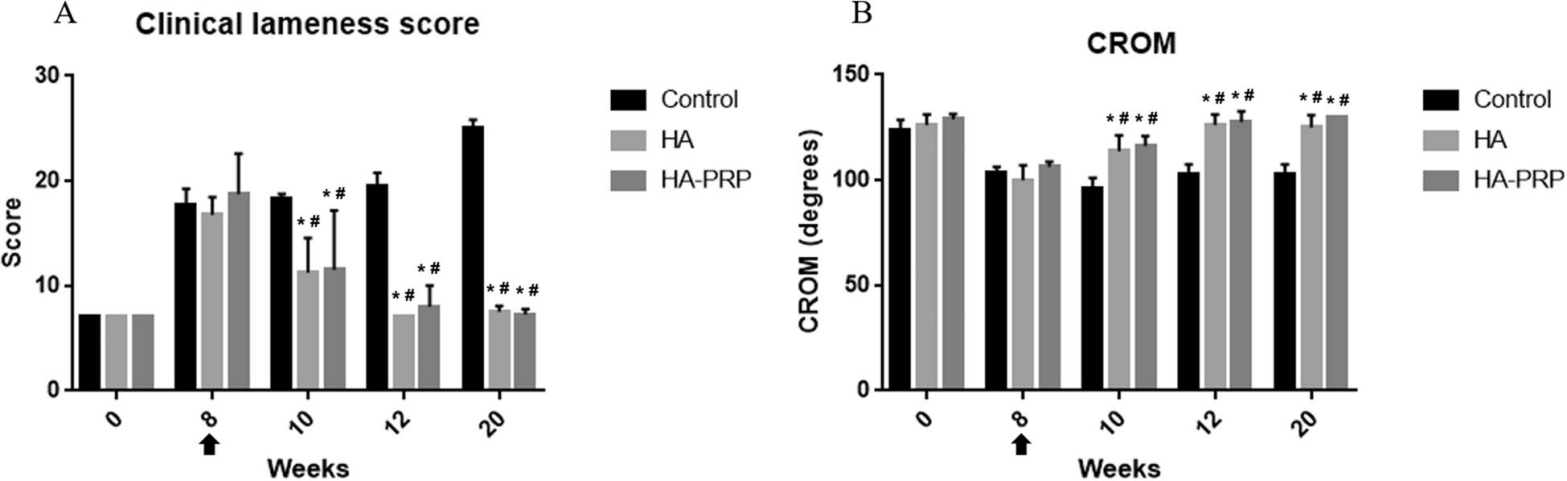
The clinical lameness score and CROM of all the experimental groups. Comparison of the values for lameness score (**a**) and CROM (**b**) in the affected knees of dogs in the saline-, HA-, and HA-PRP-treated groups over the 20-week study period. *Statistically significant difference (*p* < 0.05) compared with the control group in specific time points, and the bar represents the mean with standard deviation. ^#^Significant difference (*p* < 0.05) within group comparison over time before (8 weeks) and after (10, 12, and 20 weeks) treatment. Arrow means the time point of the first intra-articular treatment

**Fig. 2:**
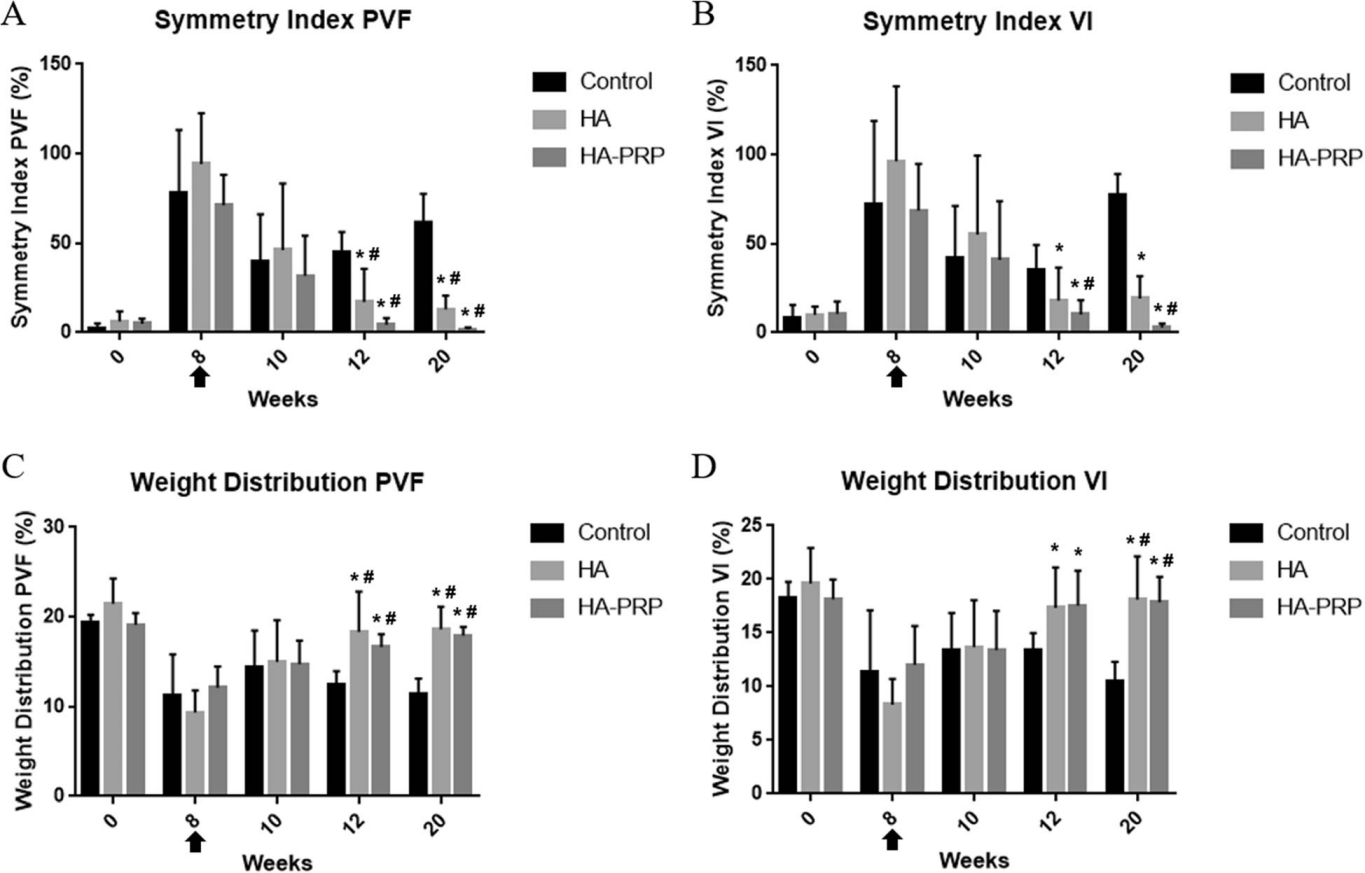
Gait analysis on pressure sensor walkway. Comparison SI and WD in the affected knees of dogs in the saline-, HA-, and HA-PRP-treated groups was over the 20-week study period. *Statistically significant difference (*p* < 0.05) compared with the control group in specific time points, and the bar represents the mean with standard deviation. ^#^Significant difference (*p* < 0.05) within group comparison over time before (8 weeks) and after (10, 12, and 20 weeks) treatment. Arrow indicates the time point of the first intra-articular treatment

Post-CrCL resection (8 weeks), all dogs were lame with an increase of difference in weight-bearing ability of bilateral hind limbs (SI) and decrease of weight distribution (WD) and CROM (Figs. 1 and 2).

In the HA and HA-PRP groups, clinical lameness scores decreased and CROM increased from 2 weeks after the first intra-articular treatment (10 weeks), whereas in the saline control group, the clinical lameness score continued to increase after the injection and the CROM remained unchanged. No significant differences in lameness (*p* > 0.05) and CROM (*p* > 0.05) were observed between the HA and HA-PRP groups over time (Fig. 1).

Symmetry and weight distribution analyses revealed improvements at weeks 12 and 20 in the two active treatment groups (HA and HA-PRP) compared to the saline control. Both active treatment groups showed a consistent trend of reduction in symmetry index differences over time. At weeks 12 and 20, the improvement was greater in the HA-PRP group than in the HA group, although this was not statistically significant. There was also no significant difference (*p* > 0.05) in weight distribution between HA and HA-PRP at any time point post-treatment (Fig. 2).

### Radiographic OA

**Fig. 3:**
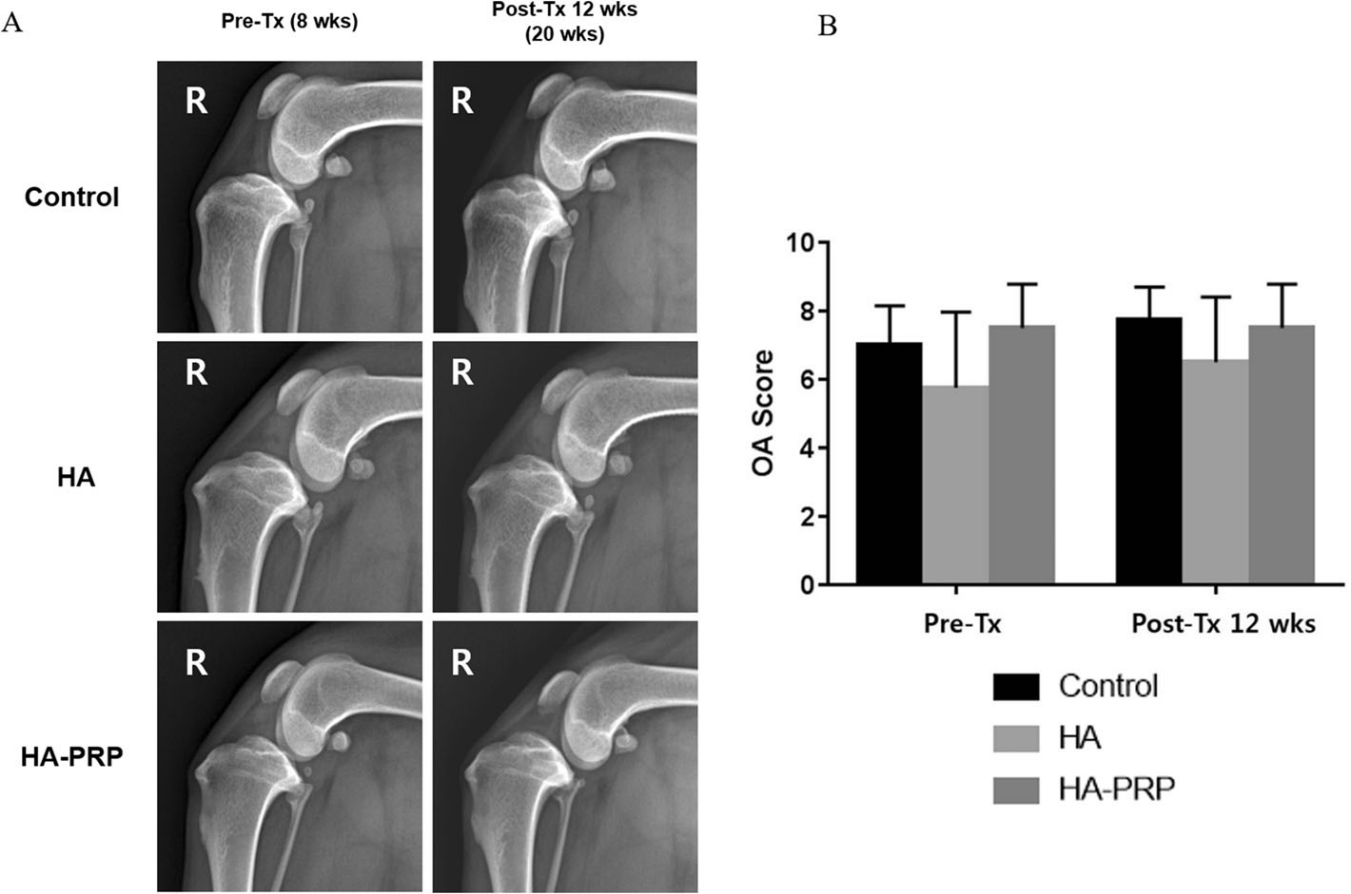
Radiographic evaluation of all dogs before and after the intra-articular injection. The radiographs in the affected knees of dogs in the control, HA, and HA-PRP groups (**a**) and comparison of the radiographic OA severity scores among three groups (**b**). No significant differences for intra-group (*p* > 0.05) OA severity score over time and inter-group (*p* > 0.05) OA severity score following the specific time points were observed

Radiographic OA severity score increased in the control and active treatment groups over the 20-week evaluation period. However, the differences in severity were not statistically significant within any group over time (*p* > 0.05) or among the groups (*p* > 0.05) at any time point (Fig. 3).

### Gross lesion assessments

**Fig. 4:**
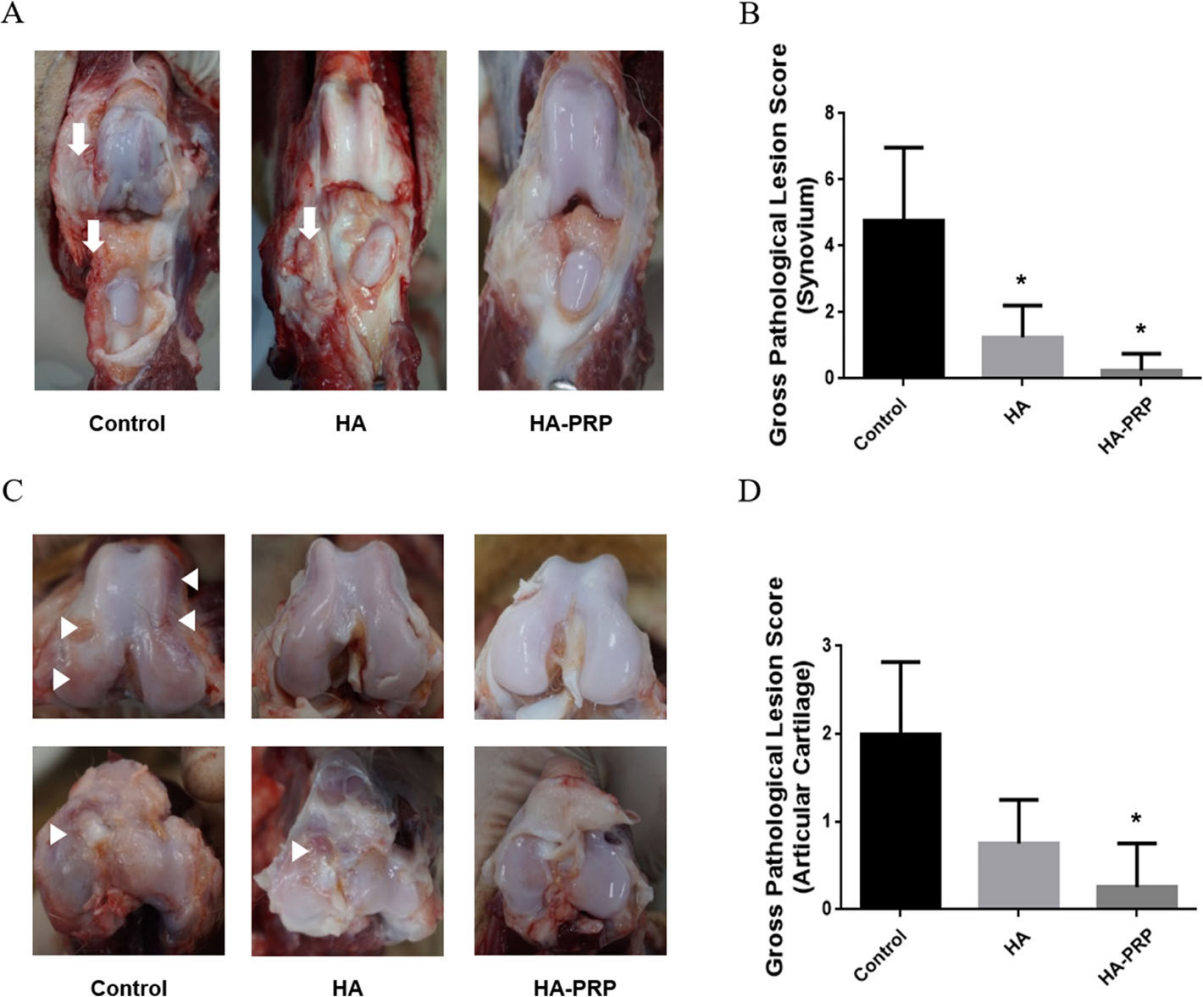
Macroscopic evaluation of the synovium and articular cartilage from all groups. Macroscopic lesions of the synovium (**a**) and articular cartilage (**c**) in the affected knees of all experimental groups. Comparison of the OARSI scores of the gross lesion in the affected synovium and articular cartilage (**b**, **d**). Proliferation and thickening with increased vascularity of the synovium were identified from the control and the HA group (**a**, white arrows). Fibrillation and roughen articular surface were observed in the weight-bearing areas of the femoral condyle and the tibial plateau (**c**, white arrowheads). *Significant difference (*p* < 0.05) between the treatment groups and the control group. OARSI scores of the synovium (*p* > 0.05) and articular cartilage (*p* > 0.05) were not significantly different when comparing between HA and HA-PRP group

Based on the gross lesion assessments of the treated stifle joints at 20 weeks after the first treatment, all groups had mild to severe articular cartilage damage (predominantly in the medial compartment), with mild to severe synovitis. Gross assessments of articular cartilage using the OARSI scoring system showed improvement in the two active treatment groups compared to control (Fig 4). Articular cartilage preservation, however, was significantly better, as evidenced by a lower OARSI mean score, only in the HA-PRP group when compared to the control saline-injected group.

### Histopathological assessments

**Fig. 5:**
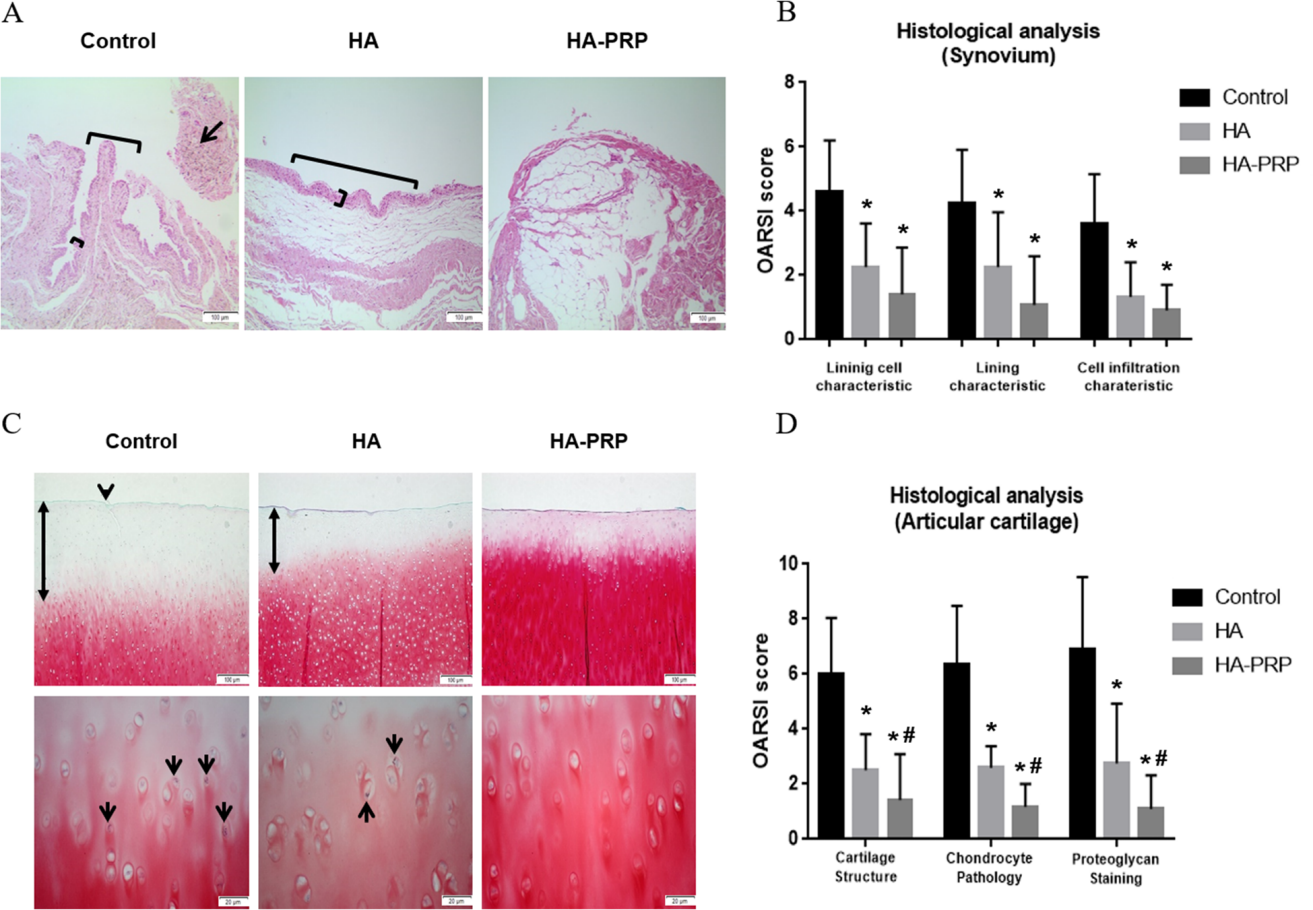
Microscopic assessments of the synovium and articular cartilage. Microscopic lesions of the synovium (**a**; H&E, scale bar = 100 μm) and articular cartilage (**c**; Safranin O, scale bar = 100 μm [upper low], scale bar = 20 μm [lower low]) in the affected knees of all experimental groups. Multiple cell layer (short arch) and villous hyperplasia (long arch) were observed in the synovium of the control and HA groups. Mild to moderate inflammatory cell infiltrates including small lymphoid follicles were identified in the control group. The articular cartilage at the superficial zone with an irregular surface (Arrowhead) was observed in the control group. The grade of proteoglycan staining (bidirectional arrows) was the lowest in the control group, followed by the HA group. The large cell clusters in chondrocyte (arrows with short body) were observed frequently compared with the HA and HA-PRP group. Comparison of the OARSI histopathologic scores in the affected synovium (**b**) and articular cartilage (**d**). *Significant differences (*p* < 0.05) compared to the groups treated with saline. ^#^Significant difference (*p* < 0.05) between the HA group and the HA-PRP group

HA and HA-PRP treatment resulted in the improvement in osteochondral lesions, as manifested on histology by a regular cartilage surface; more proteoglycan staining; and fewer chondrocyte clusters. The extent of the improvement was larger in the HA-PRP group than in the HA group. The active treatment groups had better OARSI scores by all parameters for both the articular cartilage and the synovium; however, those for articular cartilage reached significance only for the HA-PRP treated group (Fig. 5).

## Discussion

No major complication (e.g., septic arthritis) was observed following intra-articular injections in all groups during the whole period of the study. However, two dogs of the HA group showed non-weight-bearing lameness after the first injection, which was relieved within 3 days post-injection. The HA used in this study was made by fermenting *Streptococcus* species. Naturally derived HA compounds are known to result in minor local reactions like stiffness and local inflammation flare at the injection site [35]. This reaction is typically mild and self-limited, resolving within 1–3 days. One dog of the control group in this study showed systemic inflammatory reaction according to the blood work after the first injection. This may be associated with injection skill of the clinician causing destruction of the joint structure [36].

Our study provides preliminary evidence that intra-articular injections of HA and HA-PRP may result in significant improvement in subjective severity of pain and lameness scores (clinical lameness score) and CROM at 10 weeks (2 weeks after the first injection). Furthermore, at 12 weeks (4 weeks after the first treatment), a significant improvement was observed through an objective measurement of weight-bearing (SI [PVF and VI], WD [PVF and VI]) for dogs with clinical signs of OA involving a stifle joint. These data support the consideration of an intra-articular injection of HA or HA-PRP as a feasible treatment for OA in dogs.

HA used in this study has a high molecular weight (1.2 MDa). It is known that high molecular weight of HA (HMW-HA) can be maintained for a long time in the synovial space compared to the low molecular weight of HA (LMW-HA) [37]. Marshall et al. reported that the injection of HMW-HA at the knee joint 2 months after the induction of accelerated canine osteoarthritis significantly decreased the severity of the disease, based on the gross and histological features [38]. Elmorsy et al. reported less OA development, better friction coefficients, and improvement in histological scores, most notably affecting the superficial cartilage layer, when comparing HMW-HA to saline controls treated 5 weeks after cranial cruciate ligament resection [39]. Kichuchi et al. described that similar findings with HMW-HA being more effective than LMW-HA in inhibiting cartilage degradation when HA was injected immediately after meniscectomy in rabbits [40]. Pashuck et al. reported that injection of HMW-HA after 24 weeks after meniscectomy in dogs was superior to LMW-HA in amelioration of OA symptoms [30]. Our findings confirm the beneficial clinical effects of HMW-HA by both subjective and objective parameters at 10, 12, and 20 weeks compared to the saline control group including gross and histological grades of the synovium and a clear trend showing less severe articular cartilage lesions assessed grossly and histologically when compared with the control group. The current data, consistent with the results of previous studies, suggest that intra-articular injection of HMW-HA may have beneficial effects ameliorating the symptoms associated with canine osteoarthritis.

The combination of HMW-HA and PRP, and in particular the more stable product of chemical conjugation between the two components, was expected to enhance both clinical and structural benefits of HMW-HA. Based on this hypothesis, HA-PRP was injected at half the frequency of that used for HMW-HA. Using that specific protocol, there was however no statistically significant advantage for HA-PRP over that of the HA group until 20 weeks of the study, based on CROM (*p* > 0.1) and clinical lameness score (*p* > 0.7). There was, however, an advantage albeit not significant for HA-PRP over HMW-HA in gait analysis (SI of PVF [*p* > 0.2] and VI [*p* > 0.4], WD of PVF [*p* > 0.4], and VI [*p* > 0.9]), in the joint cartilage structure which was less susceptible to damage as determined by gross and histological analysis in comparison with the HA-treated group. This may be associated with the specific properties of the HA-PRP conjugate and in particular its residence time in the joint.

Several of HA derivatives have been used as hydrogel with increased residence time. Milgilore et al. and Benazzo et al. reported that HA hydrogel injection lasted the effects of pain relief for up to 26 weeks in OA patients [41, 42]. Brown and Laurent reported that intra-articular treatment of HA hydrogel could last up to 8 weeks in rabbit rheumatoid arthritis model [43]. PRP may also play an important role in the maintenance of hyaline-like chondrogenic phenotype, increase chondrocyte proliferation, and promotes proteoglycan synthesis, and as a potent chemotactic factor for all cells of mesenchymal origin. It has been reported that intra-articular injection of PRP reduce lameness score and preserve articular cartilage in groups injected with PRP compared to the control group [44]. The HA-PRP hydrogel used in the current study may have sustained the chondroprotective effect of HA in the affected joint while maintaining the articular cartilage regenerating effect of PRP. The histological data showing a clear advantage in cartilage tissue preservation suggest that HA-PRP may also alleviate the clinical signs of osteoarthritis for a longer period of time in OA patients compared to conventional HA by providing better, long-lasting protection from damage induced OA.

In a previous study using a similar protocol in rabbit femoral defect model, Liu et al. have shown that PRP was effective in improving osteoarthritis histologically and biochemically compared to the HA group [12]. Similarly, using the same injection protocol, Guler et al. has reported that PRP was more effective for alleviating clinical symptoms caused by human OA in the early stages of disease than HA [13]. In another study, OA patients who received PRP or HA with the same protocol were followed up at 3 and 6 months after treatment. It was found that pain scores were significantly lower in PRP-treated patients compared to HA-treated patients in all the time points [14]. In the present study, the HA group was given weekly injections for a total of four injections, and the HA-PRP group was injected every 2 weeks for a total of two injections. Although lower numbers of injections were performed in the HA-PRP group, similar clinical outcomes were observed when compared with the HA group. Furthermore, a histologic evaluation revealed that the articular cartilage was more preserved in the HA-PRP group than in the HA group. These findings suggest that the PRP component as well if the injections were carried out at the same frequency as in the HA group may have shown a more effective amelioration of OA symptoms.

According to radiographic assessments, none of the materials used in the current study were effective in ameliorating the development or progression of OA associated with CrCL resection in dogs. These findings correspond to Smith et al., who used intra-articular injection of HA in a CrCL resection OA model in dogs [8]. Also, Pashuck et al. described that intra-articular HA treatment was not effective to decrease the progression of OA in radiographic evaluation after meniscal releasing in dogs [30]. Based on the radiographic evaluation, no remarkable improvement in ameliorating the development of OA was found in all groups, but following the histological analysis, HA-PRP seems to be effective in improving osteoarthritis at the microscopic level.

Limitations of this study should be considered when translating these data for clinical applicability. The injection protocol was performed based on the experimental design, which is valid for pre-clinical study of OA therapeutics but does not exactly mimic the real clinical situation. There is no consensus or fully established protocols for intra-articular injection of HA in veterinary medicine, and different studies suggest different treatment regimens. One such study proposed that three weekly injection protocol is most suitable for decreasing lameness in dogs [38], while Nganvongpanit et al. suggested one or two injections in canine OA patients [45]. Establishing the correct protocol for improving OA may vary depending on the type and nature of HA. Another limitation is the relatively short follow-up of most of these studies including our current study, making it difficult to determine long-term differences, for example between HA and PRP. Long-term, large-scale clinical trials are needed in order to better establish the potential long-term beneficial effects of HA-PRP and HA used in this study.

## Conclusions

This study to the best of our knowledge is the first to compare the intra-articular injection of HA-PRP hydrogel with conventional HMW-HA for the treatment of canine OA. The results of our study suggest that HA and HA-PRP, currently used for intra-articular injections, are safe and effective in the amelioration of clinical symptoms associated with OA in dogs and HA-PRP can serve as a superior alternative treatment for long-term joint preservation in OA veterinary patients.

## Data Availability

All datasets used during the present study are available from the corresponding author on reasonable request.

## Abbreviations

CrCL: Cranial cruciate ligament
CROM: Comfortable range of motion
HA: Hyaluronic acid
HA-PRP: Hyaluronic acid conjugated with platelet-rich plasma
HMW-HA: High-molecular weight hyaluronic acid
LMW-HA: Low-molecular weight hyaluronic acid
OA: Osteoarthritis
OARSI: Osteoarthritis Research Society International
PRP: Platelet-rich plasma
PVF: Peak vertical force
SI: Symmetry index
VI: Vertical impulse
WD: Weight distribution
